# Energy Sources for Exosome Communication in a Cancer Microenvironment

**DOI:** 10.3390/cancers14071698

**Published:** 2022-03-27

**Authors:** Abhimanyu Thakur, Amanda Johnson, Emily Jacobs, Kui Zhang, Jonathan Chen, Zhubo Wei, Qizhou Lian, Huanhuan Joyce Chen

**Affiliations:** 1 The Pritzker School of Molecular Engineering, The University of Chicago, Chicago, IL 60637, USA; abhimanyu@uchicago.edu (A.T.); emilyjacobs2023@uchicago.edu (E.J.); kuizhang@uchicago.edu (K.Z.); schen77@uchicago.edu (J.C.); 2 The Ben May Department for Cancer Research, The University of Chicago, Chicago, IL 60637, USA; 3 Committee on Development, Regeneration, and Stem Cell Biology, The University of Chicago, Chicago, IL 60637, USA; amandabrooke@uchicago.edu; 4 Institute of Biosciences and Technology, Texas A&M University, Houston, TX 77030, USA; zwei@tamu.edu; 5 Department of Surgery, The University of Hong Kong Shenzhen Hospital, Shenzhen 518053, China; 6 HKUMed Laboratory of Cellular Therapeutics, and Department of Medicine, The University of Hong Kong, Hong Kong 999077, China; 7 Cord Blood Bank Center, Guangzhou Women and Children’s Medical Center, Guangzhou Medical University, Guangzhou 510180, China; 8 State Key Laboratory of Pharmaceutical Biotechnology, The University of Hong Kong, Hong Kong 999077, China

**Keywords:** exosome, extracellular vesicle, cancer, energy metabolism, tumor microenvironment

## Abstract

**Simple Summary:**

Exosomal communication in the tumor microenvironment plays a crucial role in cancer development, progression, and metastasis, and is achieved by either short-distance communication with neighboring cells or long-distance communication with distant organs. Nevertheless, how exosomes gain energy to establish such communication and the different sources of energy are unclear. Recently, a handful of studies have demonstrated the presence of mitochondria, adenosine triphosphate, and glycolytic enzymes, which may serve as potential energy sources for exosomes. This review clarifies how exosomes maintain their structural integrity and stability during their intracellular communication, and reviews evidence of their energy source.

**Abstract:**

Exosomes are crucial extracellular vesicles (EVs) with a diameter of approximately 30–200 nm. They are released by most cell types in their extracellular milieu and carry various biomolecules, including proteins and nucleic acids. Exosomes are increasingly studied in various diseases, including cancer, due to their role in local and distant cell–cell communication in which they can promote tumor growth, cancer progression, and metastasis. Interestingly, a tremendous number of exosomes is released by malignant cancer cells, and these are then taken up by autologous and heterologous recipient stromal cells such as immune cells, cancer stem cells, and endothelial cells. All these events demand an enormous amount of energy and require that exosomes remain stable while having the capacity to reach distant sites and cross physical barriers. Nevertheless, there is a dearth of research pertaining to the energy sources of exosomes, and questions remain about how they maintain their motility in the tumor microenvironment (TME) and beyond. Moreover, exosomes can produce adenosine triphosphate (ATP), an important energy molecule required by all cells, and mitochondria have been identified as one of the exosomal cargoes. These findings strengthen the prospect of exosomal communication via transfer of mitochondria and the bioenergetics of target recipient cells. In the TME, the accumulation of ATP and lactate may facilitate the entry of exosomes into cancer cells to promote metastasis, as well as help to target cancer cells at the tumor site. This review highlights how exosomes obtain sufficient energy to thrive in the TME and communicate with distant physiological destinations.

## 1. Introduction

Exosomes are nano-sized extracellular vesicles (EVs) with a diameter of approximately 30–200 nm. They carry various kinds of cargo including mRNA, proteins, lipids, long non-coding RNAs (lncRNAs) and small RNA. Exosomes are released from many different cell types following the endosomal–lysosomal pathway and are present in various biofluids including blood, cerebrospinal fluid (CSF), and urine [1,2]. It is believed that exosomes originate from multivesicular bodies (MVBs) inside the cells. The biogenesis of exosomes begins with the inward budding of the endosomal membrane, with consequent formation of MVBs. Some MVBs then fuse with the plasma membrane and release nanovesicles into the extracellular space. Apart from exosomes, there are other types of heterogenous populations of EVs, such as microvesicles (MVs) and apoptotic bodies (ABs). They can be distinguished based on their size, biogenesis pathway, and surface markers, although their sizes often overlap. MVs are around 50–1000 nm and originate directly from the cell membrane, whereas ABs are around 50–2000 nm, and are released via blebbing and fragmentation of cells undergoing apoptosis [3]. Detailed classification, as well as the characteristics of these EVs, have been discussed elsewhere [4,5]. 

Exosomes play a crucial role in various physiological and pathological events [6,7,8]. Importantly, their release is dramatically augmented under disease conditions, indicating a vital phenomenon during disease progression [9,10]. They participate in cell–cell communication by travelling to both adjacent and distant sites. However, the means by which exosomes obtain sufficient energy for communication is unclear [6]. As noted above, exosomes are released in the extracellular milieu after the fusion of MVBs with the plasma membrane, and they are required in order to overcome various energy barriers. During this event, several protein–protein and protein–lipid interactions take place that lower the energy barriers to facilitate the fusion of MVBs to the plasma membrane; for example, tethering factors, Rabs, soluble *N*-ethylmaleimide-sensitive factor attachment-protein receptors (SNAREs), and other Rab GTPases have been reported to play important roles in these interactions [11]. The role of lipids has also been investigated in the context of exosomal release. For example, hexadecylglycerol, an ether lipid precursor, stimulates the release and alters the constituents of PC-3 cell-derived exosomes, signifying that augmented cellular ether lipids are linked to the altered release of exosomes and their composition [12]. Strauss et al. demonstrated that the release of exosomes could ameliorate lysosomal storage of cholesterol in Niemann–Pick type C (NPC) disease. Free cholesterol was found to be released by exosomes and dependent on flotillin. In the presence of an over-supply of cholesterol, exosomal cholesterol release was increased in NPC disease, and could be a potential mechanism by which to overcome the cytotoxic accumulation of cholesterol in late endosome/lysosomes. This suggests that exosomes and flotillin play a role in the regulation of cholesterol homeostasis [13]. Llorente et al. revealed that when cholesterol levels were reduced in PC-3 cells by adding methyl-β-cyclodextrin (a cholesterol-sequestering agent) or by metabolically inhibiting its formation, the secretion of various exosomal proteins was augmented [14]. It is therefore clear that various proteins and lipids, and cholesterol, play crucial roles in facilitating the release of exosomes.

Accumulating evidence of glycolytic enzymes as exosomal constituents supports their potential role in providing energy to exosomes. A recent study utilizing proteomic analysis demonstrated the presence of enzymes of the glycolytic chain in exosomes and prostasomes isolated from prostate cancer cells (PC3), and both EVs could produce ATP when presented with substrates. In particular, overall extracellular ATP generation was increased for PC3-derived exosomes due to their low ATPase activity, while the production of extracellular ATP by prostasomes was relatively lower because of their high ATPase activity. The uptake of prostasomes by recipient cells, normal prostate epithelial cells (CRL2221), and PC3 cells, was glycolytic-flux-dependent and involved the generation of extracellular ATP via EVs or the production of intracellular ATP from recipient cells. Therefore, the internalization of EVs by the recipient cells can be considered an energy-demanding process, suggesting the need for an active ATPase. The ability of EVs to release extracellular ATP might play a crucial role during this process [15]. In another recent study by Ludwig et al., simultaneous inhibition of glycolysis and oxidative phosphorylation prompted a copious surge in the release of exosomes, possibly via 2′,3′-cAMP [16]. The release of exosomes from cells in the extracellular space is a complex and inadequately understood process, because until recently, they were widely dismissed as cellular waste products. Studies associated with exosomes can be facilitated via methods that enhance their generation and release. Interestingly, the treatment of cultured cells with sodium iodoacetate (IAA; glycolysis inhibitor) plus 2,4-dinitrophenol (DNP; oxidative phosphorylation inhibitor) (up to 10 µM each) has been found to augment the release of exosomes 3- to 16-fold. Moreover, in vivo injection of IAA/DNP increased the concentration of circulating exosomes and decreased the level of ATP in cells. Notably, a cell-membrane-permeable form of 2′,3′-cAMP and 3′-AMP mimicked the effects of IAA/DNP on exosome release. The effects of IAA/DNP on exosome release were augmented in cells that lacked 2′,3′-cyclic nucleotide 3′-phosphodiesterase (CNPase), an enzyme that metabolizes 2′,3′-cAMP into 2′-AMP. Therefore, the combined effect of IAA/DNP is a potent stimulus for exosome release, partly arbitrated by 2′,3′-cAMP [16]. Consequently, the role of glycolytic enzymes is believed to be crucial to the energy producing processes, including glycolysis. The roles of exosomal glycolytic enzymes warrant further investigation. 

The physical properties of exosomes may also contribute to how they communicate, travel, and acquire specificity towards target cells. One of the contributing properties is the surface charge of exosomes, mostly represented by zeta potential (ZP), a more specific electrokinetic potential that regulates nano-particle delivery [17]. In particular, the ZP of exosomes is unique because of their type, and this impacts exosome–exosome interactions. Interestingly, changes to ZP regulate how exosomes behave. For example, the tendency of exosomal aggregation can be predicted by the value of ZP, which also represents the stability and efficiency of exosomes. Additionally, the ZP of exosomes varies in different tissues and body fluids, as well as in different disease states. In the latter case, it suggests dysfunctional potential in diseases such as cancer, where exosomes are sometimes critical to disease progression [18]. 

The role of exosomes has been extensively explored in the context of normal physiology and the progression, diagnosis, and prognosis of various fatal diseases, including cancer [10,19,20,21]. This review discusses various exosomal constituents that may serve as sources of energy to overcome the energy barriers to exosomal secretion and to establish cell–cell communication. Further, the role of lipids as a source of energy for exosomes will be discussed based on the evidence acquired from glycolytic metabolons. Recently, a demonstration of the packaging and transfer of mitochondrial DNA via exosomes [22] has opened a new avenue for research. It will be discussed as a potential energy source for exosomes and a likely mechanism for delivery of ATP via exosomes. Finally, the possible connection of ZP with the stability of exosomes will be discussed, which could be a predictive marker of the potential energy of stagnant exosomes and the kinetic energy of exosomes in motion. 

## 2. Zeta Potential of Exosomes: How does Surface Charge Regulate Exosomal Behavior?

The stability of exosomes plays a crucial role in cell–cell communication, controlled by the surface charge of exosomes and often referred to as zeta potential (ZP). The ZP of dispersal systems, including emulsions, suspensions, and colloidal dispersions, is a quantitative parameter of charge stability that influences the interaction between particles. Interestingly, like other materials, exosomes acquire electrical charge on their surface upon interaction with a polar medium. Notably, a negative charge on their surface is developed when they encounter hydrophilic buffers via a mechanism involving a cascade of steps, such as differences in the electron affinity of the two phases, ionization of exosomal membrane, and the differential ion adsorption from electrolyte solutions. Importantly, the higher value of ZP yields stronger electrostatic repulsion between particles, and thereby reduces their aggregation behavior. For example, the ZP of EVs varying from −30 to +30 mV shows aggregation, even though the specific threshold of stability depends on the type of particle [18]. During exosomal communication, there is fusion of exosomes with the plasma membrane of recipient cells, with consequent expulsion of exosomal constituents. This signifies that exosome can operate as a transporter for ligands, such as the lipophilic Wnt proteins, that need a lipid environment for transport. In this scenario, exosomes serve as an optimum vehicle for carrying Wnt proteins [23]. During endocytosis or micropinocytosis-based communication, exosomal surface proteins interact with recipient cells. Eventually, exosomal surface proteins become lysed by proteases in recipient cells, producing protein fragments that may act as ligands specific to the receptors on the surface of recipient cells [24,25]. Therefore, it is evident that the specificity of exosomal communication is accomplished via interaction between the ligands and the receptors of vesicles and those of recipient cells, respectively. Notably, exosomes remain a suspension of colloid in various media, including cultured condition medium, buffer, and biofluids such as urine and serum. To safeguard their functionality, it is necessary to guarantee the dispersal of exosomes homogeneously. This will also augment their stability throughout the storage process [26]. In the present context, the value of ZP may play a crucial role in understanding and distinguishing between specific exosomal interactions and exosomal aggregation.

Conventionally, the characterization of ZP is based on a combination of measurements including streaming potentiometry, phase analysis light scattering, and Doppler velocimetry. All these methods can help to determine the electrophoretic mobility of particles in suspension. Nevertheless, the adoption of this combined approach when dealing with polydisperse samples, which could cover a wide range of ZPs, has posed a challenge. In this context, Deblois et al. demonstrated single-particle electrokinetic measurements of nanoparticles by resistive pulse sensing, based on the velocity and electrophoretic mobility of the particles [27]. Recently, tunable resistive pule sensing (TRPS) has been demonstrated to measure the ZP based on the time duration of resistive pulse signals for individual particles [28,29]. Importantly, the measurement of ZP by TRPS is robust and reproducible, as it emphasizes the duration of displacement of nanoparticles as a function of both voltage and pressure. Moreover, the ability of TRPS to measure size and ZP simultaneously depicts a novel methodology for examining the heterogenous properties of dispersed particles [27].

## 3. Long Distance and Site-Specific Transport of Exosomes in TME

EVs travel through both blood and lymph fluid to carry various types of cargo, including proteins and nucleic acids, to the body. Their destination depends on the cell of origin and its surface proteins. These small vesicles can enter cells by a variety of methods including endocytosis, phagocytosis and clathrin-coated entry [30]. How these small vesicles target cells remain largely unknown; however, it is so far accepted that there are specific ligand/receptor relationships between the cell of origin and the target [24]. This process has been the focused mode of entry for targeted drug delivery using exosomes, in addition to hijacking endocytosis [31,32]. Exosomes are a preferred mode of long-distance communication because of their ability to pass tissue barriers, and because of their targeted protein expression, enabling tumor cells to establish metastasis [33]. The development of new lymph vessels (lymph angiogenesis) promotes distal tumor formation and provides necessary nutrients to the tumor microenvironment (TME). Unlike blood vessels, cells travel through lymph networks as a result of muscle contractions or respiration [34]. Near infrared imaging (NIR) has shown that exosomes can reach the lymph nodes from the periphery within five minutes compared with hours for immune antigen-presenting cells (APCs). This is advantageous in the innate immune response because APCs are commonly needed at the original site of infection, and the rapid distribution of exosomes may facilitate a more efficient immune response. This suggests that exosomes more commonly use lymphatic transport from the periphery to lymph nodes rather than a highly selective route. In addition, exosomes have been observed to accumulate in other organs [35]. Passive transport via lymph fluid is a common mode for tumor cells. Exosomes mimic this to easily travel to distant organs, and the exosome migration facilitates the metastasis of tumor cells. Proteomic profiling has revealed that lymphatic endothelial cells (ECs) release an immense number of exosomes in response to inflammatory cytokines to augment dendritic cell (DC) migration [36]. Exosomes also possess the ability to pass many different barriers in the body, including but not limited to the blood–brain barrier (BBB), blood–retinal barrier (BRB), placental barrier (PB), and stromal barrier (SB) [20,21,37,38,39]. These important features of exosomes have made them extremely useful to recent research into drug delivery as they are highly effective at traveling through the body. The passive transport mode of EVs also means that no energy is necessary, as exosomes in high abundance can reach distant destinations.

**Figure 1:**
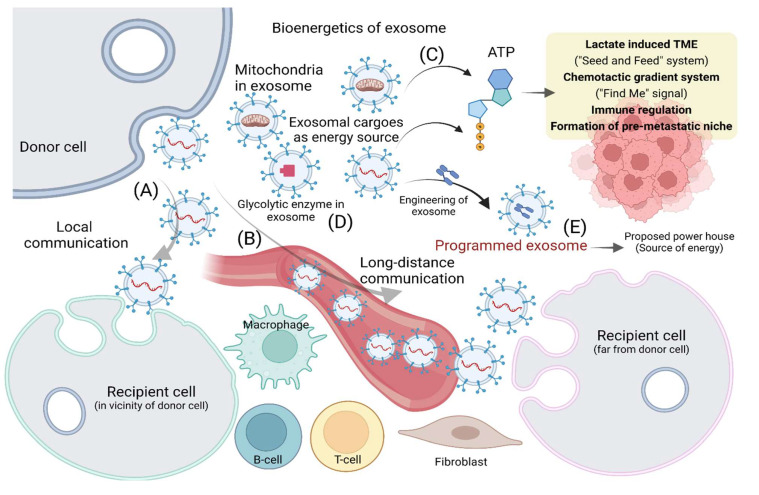
Bioenergetics of exosomes for cell–cell communication in the tumor microenvironment (TME): (**A**,**B**) local or long-distance exosomal communication in TME; (**C**,**D**) the presence of mitochondria in exosomes, the release of extracellular ATP by exosomes, and the existence of glycolytic enzymes in the proteomics of exosomes provide evidence for possible energy source; (**E**) programmed exosomes can be developed through engineering as a potential powerhouse. Figure created with BioRender.

In the context of the biodistribution of exosomes derived from culture-conditioned medium or biofluids, they can be delivered into various organs, including the liver, pancreas, spleen, kidney, colon, ovaries, and brain [40]. Nevertheless, intravenously administered exosomes mostly accumulate in the liver, followed by the lung, spleen, and gastrointestinal tract [41,42]. Intravenously injected exosomes are rapidly cleared in blood, whereas intratumoral injection facilitates longer accumulation in tumors. When administered intranasally, exosomes are predominantly delivered to the brain [43,44]. The prevailing knowledge about cell-type-specific delivery of exosomes can be found in the various aspects of exosomal motion. For example, exosomes from macrophages can be internalized by most tissues, while those from ECs have been found to be preferentially internalized in the lung [45,46,47]. In addition, the size of exosomes affects their transport; those with larger size favorably accumulate in liver, lymph nodes, and bones [48]. As noted above, although numerous studies have demonstrated the potential of exosomes as a crucial vehicle to deliver donor cell constituents to recipient cells—which may lead to phenotypic changes via exosomal communication over short or long distances (Figure 1A,B)—the motility of exosomes has not been investigated in detail. Recently, Cvjetkovic A. et al. demonstrated the presence of actin-like filaments in a subpopulation of EVs via cyro-electron micrography, suggesting that the motility of EVs was similar to that generated by actin in cells. It can be speculated that EVs have intrinsic motility, opening up a new avenue for investigation of the morphological plasticity of EVs [49]. 

## 4. Bioenergetics of Exosome Release and Uptake in TME

Donor cell release of exosomes requires a huge expenditure of both energy and biosynthetic materials. Various types of cargo of exosomes involved in bioenergetics are listed in Table 1. In particular, cancer cells release a tremendous number of exosomes compared with normal cells and regulate the immune response [50]. The generation of ATP by exosomes can be attributed to various needs such as immunoregulation and communication with stromal cells in the TME (Figure 1C). This leads to several advantages, including the production of extracellular ATP, the formation of a lactate induced TME, the establishment of a seed-and-feed system in the TME, and the creation of a long-range chemotactic gradient system or “find me” signal. ATP generated from tumor exosomes may have various effects in cancer, including regulation of the immune system. The augmented amount of ATP in the TME enhances the production of extracellular adenosine, a strong immunoregulator, and facilitates the immune evasion process in tumors [51]. The extracellular ATP has also been found to play roles in the uptake of exosomes in the TME and the communication of cancers cells with stromal cells via exosomes [15,24]. In the TME, the production of glycolytic ATP can reduce the glucose level while enhancing the lactate level, because exosomes have been found to contain lactate dehydrogenase (LDH) which facilitates the transformation of glycolytic end product pyruvate to lactate [52,53]. Another aspect of the role of extracellular ATP is that it can be taken up by tumor cells, which is energetically favorable, leading to augmented intracellular levels of ATP. This suggests that the release of ATP-generating tumor exosomes can lay the foundation for a seed-and-feed system, where cancer cells can partially outsource their energy outside the cell [54]. Extracellular ATP has also been reported as a potent “find me” signal that is employed by apoptotic bodies (a category of EVs) to attract phagocytes, leading to the formation of a chemotactic gradient in the TME [55]. In the context of cancer metastasis, the extracellular ATP produced by exosomes has also been found to create a local inflammatory state where metastasizing cells can accumulate [56,57].

## 5. How Exosomes Maintain their Structural Integrity by Overcoming Environmental Barriers

During circulation in body fluids, exosomes are confronted by an ever-changing environment. The average half-life of an EV in vivo is anticipated to be approximately 30 min, and it takes around 6 h in humans to clear intravenously injected exosomes [75]. In the human body, blood-derived exosomes move through different compartments with changing environment, suggesting that exosomes require adjustment to safeguard their structural integrity and cargo, with consequent successful delivery of functional constituents to the site of action [67]. It has been found that prostasomes inhabit various biofluids that facilitate the adherence of prostasomes to sperm, leading to the promotion of sperm fitness and motility to reach the egg [76,77]. To accomplish these tasks, exosomes possess essential and fluid-specific active surface enzymes to adapt to changing conditions [78,79,80]. Moreover, the lipid bilayer and exosomal constituents—such as chaperones, homeostasis of pH, and the optimum composition of ions—protect the exosomal cargo by preventing their aggregation. This adaptation is facilitated through ATP-driven phospholipid transporters and ion pumps, acting on the surface as well as inside the exosomes. Notably, various ATPases have been identified in the proteomics of exosomes. One such example is P2X4, a ligand-gated cation channel that has been reported to be regulated by extracellular ATP [81,82]. Owing to the presence of various ATPases and phosphatases, the level of extracellular ATP is too low in semen [83] and also low in blood plasma [84]. Consequently, the ATP-dependent proteins found in prostasomes and exosomes do not possess energy. Nevertheless, semen-derived EVs provide fructose to semen, followed by the transformation of stored glycogen to glucose to maintain an optimum level of glucose in the blood [85,86]. Therefore, fructose in semen and glucose in blood may serve as a source of energy for their respective exosomes, as well as prostasomes. Convincingly, the glycolytic production of ATP may be useful to meet crucial energy needs. 

## 6. Glycolytic Metabolons and Exosomes: An Interplay for Energy through Lipids

Glycolytic enzymes have long been found in the proteomic profile of exosomes derived from various sources (Figure 1D). Interestingly, most glycolytic enzymes have been characterized as constituents of lipid rafts [87]. The lipid element of lipid rafts shows an augmented portion of saturated fatty acid chains, offering space for cholesterols and leading to lipid domains with high order. The lipid raft domain with high orders facilitates the attraction of lipid anchors. For example, the glycosylphosphatidylinositol anchor can attach proteins with no hydrophobic membrane-spanning peptides to membranes. In addition, it also facilitates the clustering of protein on the membrane’s surface [88]. Due to these processes, the cytosolic enzymes associated with the glycolytic pathway are generally found in the lipid rafts of prostasomes [67]. Nevertheless, tetraspanin-enriched microdomains have also been found to play roles in the attachment of glycolytic enzymes to the surface. Tetraspanins create platforms to facilitate the formation of functional complexes by proteins. Importantly, tetraspanins are often found in exosomal proteomes such as CD9, C63, and CD81, that also serve as exosomal markers [89]. Notably, glycolytic enzymes identified in the lipid rafts of prostasomes include fructose-bisphosphate aldolase and other enzymes associated with the glycolytic pathway. Importantly, 6-phosphofructokinase is involved in the committed step of glycolysis, and along with hexokinase, is involved in the ATP-consuming steps. LDH has also been identified in exosomes and facilitates the conversion of pyruvate to lactate via oxidation of NADH to NAD+, and prevents the shortage of NAD+ in the subsequent glycolysis process via recycling of NAD+. Moreover, LDH has been identified in prostasomal lipid rafts [15,67]. 

**Figure 2:**
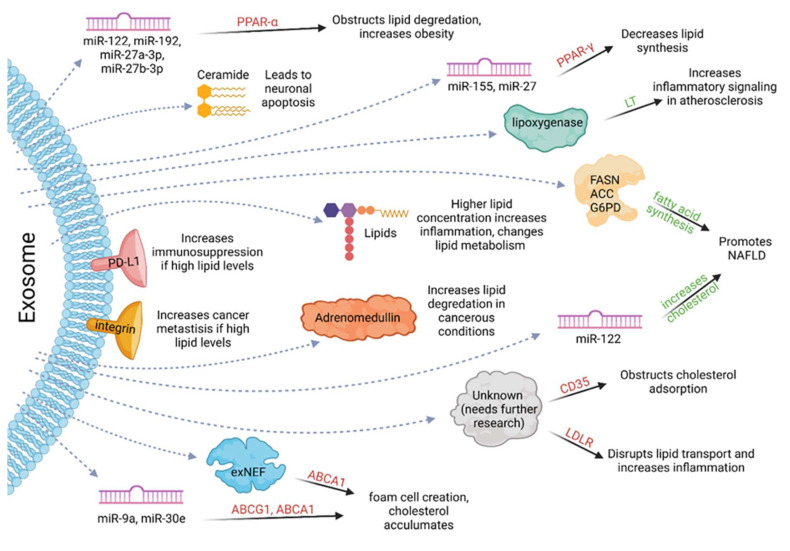
Effect of exosomal cargo on lipid metabolism. Exosomal cargo alters various types of lipid metabolism, such as lipid synthesis, degradation, and transport, which play crucial roles in regulating lipid-mediated diseases. Red text indicates that these molecules/pathways are down-regulated by the exosomal cargo, and green text indicates that these molecules/processes are up-regulated by the cargo. Figure created with BioRender.

Exosomes also play an important role in various lipid metabolism processes, such as lipid synthesis, degradation, and transport, all of which are important in regulating lipid-mediated diseases, as shown in Figure 2. Many lipid miRNAs, found as exosomal cargo, can upregulate cholesterol and fatty acid synthesis, leading to non-alcoholic fatty liver disease (NAFLD). Exosomes that carry enzymes connected with de novo lipogenesis, including ACC, FASN, and G6PD, also promote NAFLD, as they increase fatty acid synthesis. Furthermore, exosome-activated biosynthesis of leukotrienes (LTs) leads to increased atherosclerosis because LTs are pro-inflammatory. Interestingly, exosomes have also been found to decrease lipid synthesis. For example, exosomal miRNAs such as miR-122, miR-192, miR-27a-3p, and miR-27b-3p can disrupt PPAR-γ, a master controller of lipids in the liver, by binding the 3′ untranslated end of the target genes. Exosomes can also inhibit lipid transport by regulating ABCA1, ABCG1, LDLR, CD36, etc. Exosomal miRNAs can inhibit ABCA1 and ABCG1, leading to decreased transport, lipid accumulation, and foam cell creation. HIV-1 protein (exNef) displays similar effects on ABCA1, leading to the same cellular changes. Exosomes obstruct cholesterol adsorption through large decreases in macrophage CD36, although it is unknown which component within exosomes decreases CD36. Similarly, LDLR is another lipid transporter whose inhibition leads to increased inflammation; however, again, it is unknown which component within the exosome blocks LDLR. Lipid degradation can also occur through many exosomal pathways. Exosomal miRNAs can inhibit PPAR-α, leading to lower rates of lipid degradation and, thus, increased obesity. Exosomes can also carry lipids as their cargo with consequent increased inflammation and unpredictable changes in lipid metabolism. Exosomes from astrocytes can carry ceramide; when ceramide accumulates, astrocyte apoptosis occurs, specifically in Alzheimer’s patients. Exosomes also carry peptides such as adrenomedullin, or cell surface markers such as integrin or PD-L1, which promote cancer metastasis, immunosuppression, and overall disease progression [90]. Ultimately, lipids carry many important molecules that can change multiple lipid metabolic processes, leading to augmented pathogenesis of disease. There is a need to further explore these exosomal cargoes and understand how to decrease their expression and to eventually develop appropriate therapy. 

## 7. Adenosine Triphosphate as an Energy Source for EVs in Cancer Progression

Adenosine triphosphate (ATP) is deemed the molecular basis of currency in living eukaryotes, providing massive amounts of energy for cells through oxidative phosphorylation. Generated by mitochondria, ATP is synthesized when adenosine diphosphate (ADP) is turned into ATP by the addition of a phosphate molecule, driven by the electron transport chain [91]. 

Glycolysis and oxidative phosphorylation (OXPHOS) are the two foremost metabolic pathways that provide energy to cells. Glycolysis is the process of breaking down glucose into pyruvate, but is otherwise less efficient than OXPHOS at producing energy [92]. Interestingly, inflammatory immune cells utilize glycolysis more often to gain energy, a phenomenon dubbed the “Warburg Effect” [93]. This assumption is based on the knowledge that tumor cells exist in a hypoxic environment that results in a dramatic increase in glucose uptake. In this regard, although it has been believed that cancer cells almost exclusively utilize glycolysis to thrive, it has recently been suggested that they can also utilize neighboring cancer cells symbiotically. One theory posits that a cell can consume glucose and produce lactate, and a neighboring cell can obtain the secreted lactate and use it to produce ATP through OXPHOS [94]. Research is increasingly moving away from focusing only on the “Warburg effect” to looking at other co-existing energy sources such as oxidative phosphorylation [93,94]. A summary of cancer cell energy metabolism requirements from various tumor types can be found elsewhere [91].

Both glucose and lactate energy precursors in tumor cells make them heterogeneous and contribute to the complex question of how cancer cells stay metabolically active. The TME is the ecosystem of malignant tumors, and is composed of many cell types and dependent upon intercellular crosstalk [95]. Enzymes such as cycloozygenase-2 (COX-2) have been shown to be upregulated in multiple cancer types and promote metastatic progression and chemotherapy evasion [95,96]. More interestingly, increased extracellular ATP in the TME leads to increased expression of COX-2, resulting in increased invasiveness of cancer cells [96]. The TME facilitates cancer progression and metastasis by releasing circulating tumor cells (CTCs) as well as exosomes that travel from the initial tumor site; large amounts of circulating CTCs are correlated with a poor prognosis in patients [97]. Exosomes provide a paradoxical relationship within the TME, as their secretion can be initiated by both increased and decreased levels of ATP [16,98]. EVs have also been shown to provide ATP for donor cells in vitro, under both natural physiological and engineered conditions [99,100]. The release of exosomes is stimulated by P2X7 receptors that have been shown to be involved in enhancing the invasive properties of metastasizing melanoma cells to the lung, at least partially through supply of ATP [101]. This suggests an important role of ATP in the TME in the release of exosomes to instigate their journey. Kumar S et al. chemically engineered the membrane proteins of exosomes with a moiety of catechol, facilitating fusion via supramolecular complexation to bridge the membranes. Notably, this approach enabled the encapsulation of various enzymes and set up the minimal electron transport chain, consequently augmenting the catalytic reaction, enough for the synthesis of ATP. Interestingly, it was found to be functional for several hours after being internalized by recipient live cells, which showed repair of damaged regions by providing ATP. This demonstrated an excellent advancement in exosomal engineering to produce programmed exosomes as a potential energy source (Figure 1E) [100]. 

Although the travel of exosomes is largely passive through body fluids, they contain crucial energy packages that can be delivered to nearby or target cells. Exosomes do not go through replication, a common cellular process that utilizes ATP [102], but they have been shown to contain mitochondria [22,103]. Mitochondria generate power for the cell via the electron transport chain, producing the ATP needed for cell survival [104]. It is not clear if these mitochondria are required for exosomes per se, or are merely a package transfer from the cells of origin, although the latter seems more likely. Defined recently, ‘mitovesicles’ have been extracted from brain regions and appear to be mitochondria-derived small vesicles [103]. Mitochondrial dysfunction has been implicated most commonly in aging but also in cancer, where the epithelial–mesenchymal transition (EMT) is induced from this dysregulation [105]. EVs are found to contain mitochondria (Figure 1C) and mitochondrial dysfunction has been associated with these disease conditions. Neverheless, it is important to note that isolation methods vary for exosomes with different filtration sizes, so varying studies may have isolated entire mitochondria or only partial sequences. Further investigation with more cohesive isolation methods is required. 

P2X7R, an extracellular ATP receptor, is abundant in inflammatory immune cells and requires high ATP levels for activation [98]. This receptor induces the release of massive numbers of EVs in microglial cells [106]. More recently, P2X7R has been shown to be highly expressed in malignant cancer patients, correlating with a poor prognosis. The study also showed that EV miRNA content was highly dependent on P2X7R expression, which was altered by varying ATP levels [101]. In both mouse organ cultures and EC cultures, lower ATP levels caused by inhibited glycolysis enhanced the secretion of EVs [16]. Taken together, this shows a complicated, dynamic but paradoxical relationship of ATP with EVs. Applying this hypothesis, the different states of the cells of origin may account for the up- and down-regulation of ATP in EV secretion. Overall, it also suggests that EVs do not explicitly need ATP to be activated, but further detailed investigation is needed before any conclusions can be drawn.

## 8. Conclusions and Future Remarks

The multifaceted roles of exosomes—including being cell–cell communication agents, delivery vehicles, and diagnostic or prognostic platforms—have attracted much attention in the context of both physiological and pathological conditions. In addition, the tremendous release of exosomes under pathological conditions such as cancer implicates them as a remarkable biophysical marker. After release, exosomes are internalized by various recipient cells that may be close or far from the cells of origin, compelling us to wonder how these EVs obtain sufficient energy for transportation. Moreover, in the past, little research has explored the bioenergetics of exosomes required for their crucial functions. Here, the finding that glycolytic enzymes exist in the constituents of exosomes has been discussed in the context of their potential role in supplying energy to exosomes, inferring that the glycolytic metabolon of exosomes could be an energy source. Another important aspect of exosomes, which has been discovered recently, is that they produce ATP. This could be credited to various crucial functions, such as the immune regulation and facilitation of exosomal communication with surrounding cells in the TME. The extracellular ATP produced by exosomes has been shown to promote the lactate-induced “seed and feed” system in the TME and act as a “find me” signal for establishing a long-range chemotactic gradient. 

During circulation, the lipid bilayer and exosomal constituents, including auxiliary proteins, pH homeostasis, and ion composition, preserve the integrity of the exosomal structure and its cargo. Although the primary means of exosomal transport is passive via biofluids, they have been found to carry important energy packages of mitochondria that can be delivered to target recipient cells. In general, mitochondria produce power for the cell through the electron transport chain by generating ATP. Nevertheless, further investigation is needed to determine if these mitochondria are necessary for the exosomes themselves, or if they are merely exosome cargo derived from certain donor cells. Additionally, among many factors, the stability of exosomes is crucial for the successful delivery of their cargo from donor cells to recipient cells. The stability of exosomes has also been linked with their aggregation behavior. The surface charge of exosomes, represented by ZP, has been found to control exosomal stability and affect cell–cell communication; this implies that ZP can facilitate further study of exosomal behavior and differentiate between precise exosomal interaction and aggregation. 

Despite a plethora of research into exosomes, much more is still needed to investigate the bioenergetics of exosomes and other EVs. The precise measurement of energy expenditure during various exosomal processes, such as biogenesis, release, and uptake, may be new avenues for exosomal research. Additionally, further understanding of the bioenergetic mechanisms of each function of the exosome can be pursued to better understand not only how much energy is used, but how and why it is used. The investigation of the implications of higher- versus lower-energy exosomes in different disease states, and their potential relation with cancer progress, might reveal new targets for exosomal energy levels to control certain diseases. The reprogramming of exosomes via their engineering to mimic cellular features, including the generation of ATP, may pave the way for artificial sources of energy that, in turn, can be translated clinically into a new approach to nano biomedicine.

## Figures and Tables

**Table 1:** Exosomal cargo involved in bioenergetics.

Type of Cargo	Exosomal Cargo	Donor Cells	Metabolic Process	Ref.
Protein	Pyruvate kinase M2 (PKM2), Glucose transporter 1 (GLUT1)	Human hepatic stellate cell (LX-2), primary hematopoietic stem cells (HSCs)	Glycolysis induction	[58]
	Aldolase A (ALDOA), Aldehyde Dehydrogenase 3 Family Member A1 (ALDH3A1)	Lung cancer cells (A549 and NCI-H446)	Glycolysis induction	[59]
	Latent membrane protein 1 (LMP1)	CNEI-LMP1(CM) is a stable LMP1-integrated cell	Glycolysis	[60]
	Integrin beta 4 (ITGB4)	Breats cancer cells (MDA-MB-231, BT-20)	Glycolysis	[61]
	Fatty acid oxidation (FAO)-related proteins	Adipocytes	Fatty acid oxidation	[62]
	Adrenomedullin (AM)	Prostate cancer (PC) patient-derived cell lines	Lipolysis induction	[63]
	Monocarboxylate transporter 1 (MCT1), Cluster of differentiation 147 (CD147)	Glioma cells	Metabolic reprogramming	[20]
	Glucose-6-phosphate dehydrogenase (G6PDH)	Osteosarcoma cells	Pentose phosphate pathway	[64]
	Transketolase	Osteosarcoma cells	Pentose phosphate pathway	[64]
	Transaldolase 1	Osteosarcoma cells	Pentose phosphate pathway	[64]
	Glucose transporter 1 (GLUT1)	Colorectal cancer cells	Glycolysis	[65]
	Arginase-1	Ovarian cancer cells	Amino acid metabolism	[66]
Enzymes	Hexokinase, Glucose-6-phosphate isomerase 6-Phosphofructokinase (PFKL), Fructose-bisphosphate aldolase, Triosephosphate isomerase, Glyceraldehyde-3-phosphate dehydrogenase (GAPDH), Phosphoglycerate kinase (PGK), Phosphoglycerate mutase (PGM), Enolase, Pyruvate kinase, Lactate dehydrogenase-A (LDH-A)	Prostate cancer cells	Glycolysis	[67]
lncRNA	Small Nucleolar RNA Host Gene 3 (SNHG3)	Breast cancer patient-derived fibroblast cells	Metabolic reprogramming	[68]
	HIF-1α-stabilizing long noncoding RNA (HISLA)	TAMs	Glycolysis	[69]
miRNA	miR-105	Cancer-associated fibroblasts	Glycolysis	[70]
	miR-122	Human mammary epithelial cell (MCF10A) and MDA-MB-231	Metabolic reprogramming	[71]
	miR-155 miR-210	Melanoma cells	Glycolysis, Metabolic reprogramming	[72]
circRNA	ciRS-122	SW480/oxaliplatin (L-OHP) and HCT116/L-OH	Glycolysis	[73]
Metabolite	Lactate, Glutamate	Human mesenchymal stem cells(MSCs)	Amino acid metabolism	[74]

Note: exosomal cargoes—the enriched composition of exosomes; donor cells—cells from which exosomes originate.

## References

[1] Qiu G., Thakur A., Xu C., Ng S.-P., Lee Y., Wu C.-M.L. (2019). Detection of Glioma-Derived Exosomes with the Biotinylated Antibody-Functionalized Titanium Nitride Plasmonic Biosensor. Adv. Funct. Mater..

[2] Thakur A., Qiu G., NG S.-P., Guan J., Yue J., Lee Y., Wu C.-M.L. (2017). Direct detection of two different tumor-derived extracellular vesicles by SAM-AuNIs LSPR biosensor. Biosens. Bioelectron..

[3] Willms E., Cabañas C., Mäger I., Wood M.J.A., Vader P. (2018). Extracellular Vesicle Heterogeneity: Subpopulations, Isolation Techniques, and Diverse Functions in Cancer Progression. Front. Immunol..

[4] Thakur A., Parra D.C., Motallebnejad P., Brocchi M., Chen H.J. (2022). Exosomes: Small vesicles with big roles in cancer, vaccine development, and therapeutics. Bioact. Mater..

[5] Thakur A., Ke X., Chen Y.-W., Motallebnejad P., Zhang K., Lian Q., Chen H.J. (2021). The mini player with diverse functions: Extracellular vesicles in cell biology, disease, and therapeutics. Protein Cell.

[6] Keller S., Sanderson M.P., Stoeck A., Altevogt P. (2006). Exosomes: From biogenesis and secretion to biological function. Immunol. Lett..

[7] Thakur A., Qiu G., NG S.-P., Wu C.-M.L., Lee Y. (2017). Detection of membrane antigens of extracellular vesicles by surface plasmon resonance. J. Lab. Precis. Med..

[8] Thakur A. (2021). Nano therapeutic approaches to combat progression of metastatic prostate cancer. Adv. Cancer Biology-Metastasis.

[9] Naureen J., Debabrata M. (2017). Exosomes and their role in the micro-/macro-environment: A comprehensive review. J. Biomed. Res..

[10] Thakur A., Xu C., Li W.K., Qiu G., He B., Ng S.-P., Wu C.-M.L., Lee Y. (2021). In vivo liquid biopsy for glioblastoma malignancy by the AFM and LSPR based sensing of exosomal CD44 and CD133 in a mouse model. Biosens. Bioelectron..

[11] Pfeffer S.R. (2007). Unsolved Mysteries in Membrane Traffic. Annu. Rev. Biochem..

[12] Phuyal S., Skotland T., Hessvik N.P., Simolin H., Øverbye A., Brech A., Parton R.G., Ekroos K., Sandvig K., Llorente A. (2015). The Ether Lipid Precursor Hexadecylglycerol Stimulates the Release and Changes the Composition of Exosomes Derived from PC-3 Cells. J. Biol. Chem..

[13] Strauss K., Goebel C., Runz H., Möbius W., Weiss S., Feussner I., Simons M., Schneider A. (2010). Exosome Secretion Ameliorates Lysosomal Storage of Cholesterol in Niemann-Pick Type C Disease. J. Biol. Chem..

[14] Llorente A., van Deurs B., Sandvig K. (2007). Cholesterol regulates prostasome release from secretory lysosomes in PC-3 human prostate cancer cells. Eur. J. Cell Biol..

[15] Ronquist K.G., Sanchez C., Dubois L., Chioureas D., Fonseca P., Larsson A., Ullén A., Yachnin J., Ronquist G., Panaretakis T. (2016). Energy-requiring uptake of prostasomes and PC3 cell-derived exosomes into non-malignant and malignant cells. J. Extracell. Vesicles.

[16] Ludwig N., Yerneni S.S., Menshikova E.V., Gillespie D.G., Jackson E.K., Whiteside T.L. (2020). Simultaneous Inhibition of Glycolysis and Oxidative Phosphorylation Triggers a Multi-Fold Increase in Secretion of Exosomes: Possible Role of 2′,3′-cAMP. Sci. Rep..

[17] Honary S., Zahir F. (2013). Effect of Zeta Potential on the Properties of Nano-Drug Delivery Systems—A Review (Part 1). Trop. J. Pharm. Res..

[18] Beit-Yannai E., Tabak S., Stamer W.D. (2018). Physical exosome:exosome interactions. J. Cell. Mol. Med..

[19] Xu C., Thakur A., Li Z., Yang T., Zhao C., Li Y., Lee Y., Wu C.-M.L. (2021). Determination of glioma cells’ malignancy and their response to TMZ via detecting exosomal BIGH3 by a TiO2-CTFE-AuNIs plasmonic biosensor. Chem. Eng. J..

[20] Thakur A., Qiu G., Xu C., Han X., Yang T., NG S.P., Chan K.W.Y., Wu C.M.L., Lee Y. (2020). Label-free sensing of exosomal MCT1 and CD147 for tracking metabolic reprogramming and malignant progression in glioma. Sci. Adv..

[21] Thakur A., Sidu R.K., Zou H., Alam M.K., Yang M., Lee Y. (2020). Inhibition of Glioma Cells’ Proliferation by Doxorubicin-Loaded Exosomes via Microfluidics. Int. J. Nanomed..

[22] Sansone P., Savini C., Kurelac I., Chang Q., Amato L.B., Strillacci A., Stepanova A., Iommarini L., Mastroleo C., Daly L. (2017). Packaging and transfer of mitochondrial DNA via exosomes regulate escape from dormancy in hormonal therapy-resistant breast cancer. Proc. Natl. Acad. Sci. USA.

[23] Gross J.C., Chaudhary V., Bartscherer K., Boutros M. (2012). Active Wnt proteins are secreted on exosomes. Nat. Cell Biol..

[24] Mulcahy L.A., Pink R.C., Carter D.R.F. (2014). Routes and mechanisms of extracellular vesicle uptake. J. Extracell. Vesicles.

[25] Mathivanan S., Ji H., Simpson R.J. (2010). Exosomes: Extracellular organelles important in intercellular communication. J. Proteom..

[26] Hood J.L., Scott M.J., Wickline S.A. (2014). Maximizing exosome colloidal stability following electroporation. Anal. Biochem..

[27] Vogel R., Pal A.K., Jambhrunkar S., Patel P., Thakur S.S., Reátegui E., Parekh H.S., Saá P., Stassinopoulos A., Broom M.F. (2017). High-Resolution Single Particle Zeta Potential Characterisation of Biological Nanoparticles using Tunable Resistive Pulse Sensing. Sci. Rep..

[28] Blundell E.L.C.J., Vogel R., Platt M. (2016). Particle-by-Particle Charge Analysis of DNA-Modified Nanoparticles Using Tunable Resistive Pulse Sensing. Langmuir.

[29] Sikora A., Shard A.G., Minelli C. (2016). Size and ζ-Potential Measurement of Silica Nanoparticles in Serum Using Tunable Resistive Pulse Sensing. Langmuir.

[30] Tian T., Zhu Y.-L., Zhou Y.-Y., Liang G.-F., Wang Y.-Y., Hu F.-H., Xiao Z.-D. (2014). Exosome Uptake through Clathrin-mediated Endocytosis and Macropinocytosis and Mediating miR-21 Delivery. J. Biol. Chem..

[31] Tian T., Zhang H.-X., He C.-P., Fan S., Zhu Y.-L., Qi C., Huang N.-P., Xiao Z.-D., Lu Z.-H., Tannous B.A. (2018). Surface functionalized exosomes as targeted drug delivery vehicles for cerebral ischemia therapy. Biomaterials.

[32] Gaurav I., Thakur A., Iyaswamy A., Wang X., Chen X., Yang Z. (2021). Factors Affecting Extracellular Vesicles Based Drug Delivery Systems. Molecules.

[33] Park R.J., Hong Y.J., Wu Y., Kim P.M., Hong Y.-K. (2018). Exosomes as a Communication Tool Between the Lymphatic System and Bladder Cancer. Int. Neurourol. J..

[34] Christiansen A., Detmar M. (2011). Lymphangiogenesis and Cancer. Genes Cancer.

[35] Srinivasan S., Vannberg F.O., Dixon J.B. (2016). Lymphatic transport of exosomes as a rapid route of information dissemination to the lymph node. Sci. Rep..

[36] Brown M., Johnson L.A., Leone D.A., Majek P., Vaahtomeri K., Senfter D., Bukosza N., Schachner H., Asfour G., Langer B. (2018). Lymphatic exosomes promote dendritic cell migration along guidance cues. J. Cell Biol..

[37] Elliott R.O., He M. (2021). Unlocking the Power of Exosomes for Crossing Biological Barriers in Drug Delivery. Pharmaceutics.

[38] Park J., Zhang Y., Vykhodtseva N., Akula J.D., McDannold N.J. (2012). Targeted and Reversible Blood-Retinal Barrier Disruption via Focused Ultrasound and Microbubbles. PLoS ONE.

[39] Thakur A., Zou H., Yang M., Lee Y. (2018). Abstract 3720: Augmented loading efficiency of doxorubicin into glioma-derived exosomes by an integrated microfluidic device. Cancer Res..

[40] Morishita M., Takahashi Y., Nishikawa M., Takakura Y. (2017). Pharmacokinetics of Exosomes—An Important Factor for Elucidating the Biological Roles of Exosomes and for the Development of Exosome-Based Therapeutics. J. Pharm. Sci..

[41] Murphy D.E., de Jong O.G., Brouwer M., Wood M.J., Lavieu G., Schiffelers R.M., Vader P. (2019). Extracellular vesicle-based therapeutics: Natural versus engineered targeting and trafficking. Exp. Mol. Med..

[42] Munagala R., Aqil F., Jeyabalan J., Gupta R.C. (2016). Bovine milk-derived exosomes for drug delivery. Cancer Lett..

[43] Smyth T., Kullberg M., Malik N., Smith-Jones P., Graner M.W., Anchordoquy T.J. (2015). Biodistribution and delivery efficiency of unmodified tumor-derived exosomes. J. Control. Release.

[44] Zhuang X., Xiang X., Grizzle W., Sun D., Zhang S., Axtell R.C., Ju S., Mu J., Zhang L., Steinman L. (2011). Treatment of Brain Inflammatory Diseases by Delivering Exosome Encapsulated Anti-inflammatory Drugs From the Nasal Region to the Brain. Mol. Ther..

[45] Kim S.H., Bianco N.R., Shufesky W.J., Morelli A.E., Robbins P.D. (2007). Effective Treatment of Inflammatory Disease Models with Exosomes Derived from Dendritic Cells Genetically Modified to Express IL-4. J. Immunol..

[46] Chow A., Zhou W., Liu L., Fong M.Y., Champer J., Van Haute D., Chin A.R., Ren X., Gugiu B.G., Meng Z. (2015). Macrophage immunomodulation by breast cancer-derived exosomes requires Toll-like receptor 2-mediated activation of NF-κB. Sci. Rep..

[47] Charoenviriyakul C., Takahashi Y., Morishita M., Matsumoto A., Nishikawa M., Takakura Y. (2017). Cell type-specific and common characteristics of exosomes derived from mouse cell lines: Yield, physicochemical properties, and pharmacokinetics. Eur. J. Pharm. Sci..

[48] Zhang H., Freitas D., Kim H.S., Fabijanic K., Li Z., Chen H., Mark M.T., Molina H., Martin A.B., Bojmar L. (2018). Identification of distinct nanoparticles and subsets of extracellular vesicles by asymmetric flow field-flow fractionation. Nat. Cell Biol..

[49] Cvjetkovic A., Crescitelli R., Lässer C., Zabeo D., Widlund P., Nyström T., Höög J., Lötvall J. (2017). Extracellular vesicles in motion. Matters.

[50] Morrissey S.M., Zhang F., Ding C., Montoya-Durango D.E., Hu X., Yang C., Wang Z., Yuan F., Fox M., Zhang H. (2021). Tumor-derived exosomes drive immunosuppressive macrophages in a pre-metastatic niche through glycolytic dominant metabolic reprogramming. Cell Metab..

[51] Clayton A., Al-Taei S., Webber J., Mason M.D., Tabi Z. (2011). Cancer Exosomes Express CD39 and CD73, Which Suppress T Cells through Adenosine Production. J. Immunol..

[52] Robergs R.A., Ghiasvand F., Parker D. (2004). Biochemistry of exercise-induced metabolic acidosis. Am. J. Physiol. Integr. Comp. Physiol..

[53] Husain Z., Seth P., Sukhatme V. (2013). Tumor-derived lactate and myeloid-derived suppressor cells: Linking metabolism to cancer immunology. Oncoimmunology.

[54] Qian Y., Wang X., Li Y., Cao Y., Chen X. (2016). Extracellular ATP a New Player in Cancer Metabolism: NSCLC Cells Internalize ATP In Vitro and In Vivo Using Multiple Endocytic Mechanisms. Mol. Cancer Res..

[55] Ravichandran K.S. (2010). Find-me and eat-me signals in apoptotic cell clearance: Progress and conundrums. J. Exp. Med..

[56] Sceneay J., Smyth M.J., Möller A. (2013). The pre-metastatic niche: Finding common ground. Cancer Metastasis Rev..

[57] Hood J.L., San R.S., Wickline S.A. (2011). Exosomes Released by Melanoma Cells Prepare Sentinel Lymph Nodes for Tumor Metastasis. Cancer Res..

[58] Wan L., Xia T., Du Y., Liu J., Xie Y., Zhang Y., Guan F., Wu J., Wang X., Shi C. (2019). Exosomes from activated hepatic stellate cells contain GLUT1 and PKM2: A role for exosomes in metabolic switch of liver nonparenchymal cells. FASEB J..

[59] Wang C., Xu J., Yuan D., Bai Y., Pan Y., Zhang J., Shao C. (2020). Exosomes carrying ALDOA and ALDH3A1 from irradiated lung cancer cells enhance migration and invasion of recipients by accelerating glycolysis. Mol. Cell. Biochem..

[60] Wu X., Zhou Z., Xu S., Liao C., Chen X., Li B., Peng J., Li D., Yang L. (2020). Extracellular vesicle packaged LMP1-activated fibroblasts promote tumor progression via autophagy and stroma-tumor metabolism coupling. Cancer Lett..

[61] Sung J.S., Kang C.W., Kang S., Jang Y., Chae Y.C., Kim B.G., Cho N.H. (2020). ITGB4-mediated metabolic reprogramming of cancer-associated fibroblasts. Oncogene.

[62] Lazar I., Clement E., Dauvillier S., Milhas D., Ducoux-Petit M., LeGonidec S., Moro C., Soldan V., Dalle S., Balor S. (2016). Adipocyte Exosomes Promote Melanoma Aggressiveness through Fatty Acid Oxidation: A Novel Mechanism Linking Obesity and Cancer. Cancer Res..

[63] Sagar G., Sah R.P., Javeed N., Dutta S.K., Smyrk T.C., Lau J.S., Giorgadze N., Tchkonia T., Kirkland J.L., Chari S.T. (2016). Pathogenesis of pancreatic cancer exosome-induced lipolysis in adipose tissue. Gut.

[64] Shen R., Zhu X., Yi H., Wu C., Chen F., Dai L., Lin J. (2016). Proteomic identification of osteosarcoma-derived exosomes and their activation of pentose phosphate pathway. Int. J. Clin. Exp. Pathol..

[65] Preet R., Dixon D.A. (2018). Mutant KRAS Exosomes Influence the Metabolic State of the Colon Microenvironment. Cell. Mol. Gastroenterol. Hepatol..

[66] Sosnowska A., Czystowska-Kuzmicz M., Golab J. (2019). Extracellular vesicles released by ovarian carcinoma contain arginase 1 that mitigates antitumor immune response. Oncoimmunology.

[67] Göran Ronquist K. (2019). Extracellular vesicles and energy metabolism. Clin. Chim. Acta.

[68] Li Y., Zhao Z., Liu W., Li X. (2020). SNHG3 Functions as miRNA Sponge to Promote Breast Cancer Cells Growth Through the Metabolic Reprogramming. Appl. Biochem. Biotechnol..

[69] Chen F., Chen J., Yang L., Liu J., Zhang X., Zhang Y., Tu Q., Yin D., Lin D., Wong P.-P. (2019). Extracellular vesicle-packaged HIF-1α-stabilizing lncRNA from tumour-associated macrophages regulates aerobic glycolysis of breast cancer cells. Nat. Cell Biol..

[70] Yan W., Wu X., Zhou W., Fong M.Y., Cao M., Liu J., Liu X., Chen C.-H., Fadare O., Pizzo D.P. (2018). Cancer-cell-secreted exosomal miR-105 promotes tumour growth through the MYC-dependent metabolic reprogramming of stromal cells. Nat. Cell Biol..

[71] Fong M.Y., Zhou W., Liu L., Alontaga A.Y., Chandra M., Ashby J., Chow A., O’Connor S.T.F., Li S., Chin A.R. (2015). Breast-cancer-secreted miR-122 reprograms glucose metabolism in premetastatic niche to promote metastasis. Nat. Cell Biol..

[72] Shu S.L., Yang Y., Allen C.L., Maguire O., Minderman H., Sen A., Ciesielski M.J., Collins K.A., Bush P.J., Singh P. (2018). Metabolic reprogramming of stromal fibroblasts by melanoma exosome microRNA favours a pre-metastatic microenvironment. Sci. Rep..

[73] Wang X., Zhang H., Yang H., Bai M., Ning T., Deng T., Liu R., Fan Q., Zhu K., Li J. (2020). Exosome-delivered circRNA promotes glycolysis to induce chemoresistance through the miR-122-PKM2 axis in colorectal cancer. Mol. Oncol..

[74] Vallabhaneni K.C., Penfornis P., Dhule S., Guillonneau F., Adams K.V., Mo Y.Y., Xu R., Liu Y., Watabe K., Vemuri M.C. (2015). Extracellular vesicles from bone marrow mesenchymal stem/stromal cells transport tumor regulatory microRNA, proteins, and metabolites. Oncotarget.

[75] Lai C.P., Mardini O., Ericsson M., Prabhakar S., Maguire C.A., Chen J.W., Tannous B.A., Breakefield X.O. (2014). Dynamic Biodistribution of Extracellular Vesicles in Vivo Using a Multimodal Imaging Reporter. ACS Nano.

[76] Stegmayr B., Ronquist G. (1982). Promotive effect on human sperm progressive motility by prostasomes. Urol. Res..

[77] Saez F. (2016). Prostasomes post-testicular sperm maturation and fertility. Front. Biosci..

[78] Ronquist G.K., Larsson A., Stavreus-Evers A., Ronquist G. (2012). Prostasomes are heterogeneous regarding size and appearance but affiliated to one DNA-containing exosome family. Prostate.

[79] Valadi H., Ekström K., Bossios A., Sjöstrand M., Lee J.J., Lötvall J.O. (2007). Exosome-mediated transfer of mRNAs and microRNAs is a novel mechanism of genetic exchange between cells. Nat. Cell Biol..

[80] Ronquist K.G., Ronquist G., Carlsson L., Larsson A. (2009). Human prostasomes contain chromosomal DNA. Prostate.

[81] Kawate T., Michel J.C., Birdsong W.T., Gouaux E. (2009). Crystal structure of the ATP-gated P2X4 ion channel in the closed state. Nature.

[82] Pisitkun T., Shen R.-F., Knepper M.A. (2004). Identification and proteomic profiling of exosomes in human urine. Proc. Natl. Acad. Sci. USA.

[83] Gottlieb C., Svanborg K., Eneroth P., Bygdeman M. (1987). Adenosine triphosphate in human semen: A study on conditions for a bioluminescence assay**Supported by the Swedish Medical Research Council, Project no. B85-17X-05696-06A, and the World Health Organization Task Force on Infertility, Geneva, Switzerland. Fertil. Steril..

[84] Harkness R.A., Coade S.B., Webster A.D.B. (1984). ATP, ADP and AMP in plasma from peripheral venous blood. Clin. Chim. Acta.

[85] Schirren C. (1963). Relation between fructose content of semen and fertility in Man. Reproduction.

[86] Wasserman D.H. (2009). Four grams of glucose. Am. J. Physiol. Metab..

[87] Dubois L., Ek B., Ronquist G., Larsson A. (2015). Proteomic Profiling of Detergent Resistant Membranes (Lipid Rafts) of Prostasomes. Mol. Cell. Proteom..

[88] Simons K., Ikonen E. (1997). Functional rafts in cell membranes. Nature.

[89] Yáñez-Mó M., Barreiro O., Gordon-Alonso M., Sala-Valdés M., Sánchez-Madrid F. (2009). Tetraspanin-enriched microdomains: A functional unit in cell plasma membranes. Trends Cell Biol..

[90] Wang W., Zhu N., Yan T., Shi Y.-N., Chen J., Zhang C.-J., Xie X.-J., Liao D.-F., Qin L. (2020). The crosstalk: Exosomes and lipid metabolism. Cell Commun. Signal..

[91] Moreno-Sánchez R., Rodríguez-Enríquez S., Marín-Hernández A., Saavedra E. (2007). Energy metabolism in tumor cells. FEBS J..

[92] Wang Z., Guan D., Wang S., Chai L.Y.A., Xu S., Lam K.-P. (2020). Glycolysis and Oxidative Phosphorylation Play Critical Roles in Natural Killer Cell Receptor-Mediated Natural Killer Cell Functions. Front. Immunol..

[93] Liberti M.V., Locasale J.W. (2016). The Warburg Effect: How Does it Benefit Cancer Cells?. Trends Biochem. Sci..

[94] Sonveaux P., Végran F., Schroeder T., Wergin M.C., Verrax J., Rabbani Z.N., De Saedeleer C.J., Kennedy K.M., Diepart C., Jordan B.F. (2008). Targeting lactate-fueled respiration selectively kills hypoxic tumor cells in mice. J. Clin. Investig..

[95] Fiebich B.L., Akter S., Akundi R.S. (2014). The two-hit hypothesis for neuroinflammation: Role of exogenous ATP in modulating inflammation in the brain. Front. Cell. Neurosci..

[96] Ferrandina G., Lauriola L., Zannoni G.F., Fagotti A., Fanfani F., Legge F., Maggiano N., Gessi M., Mancuso S., Ranelletti F.O. (2002). Increased cyclooxygenase-2 (COX-2) expression is associated with chemotherapy resistance and outcome in ovarian cancer patients. Ann. Oncol..

[97] Balkwill F.R., Capasso M., Hagemann T. (2012). The tumor microenvironment at a glance. J. Cell Sci..

[98] Lombardi M., Gabrielli M., Adinolfi E., Verderio C. (2021). Role of ATP in Extracellular Vesicle Biogenesis and Dynamics. Front. Pharmacol..

[99] Dave K.M., Zhao W., Hoover C., D’Souza A., Manickam D.S. (2021). Extracellular Vesicles Derived from a Human Brain Endothelial Cell Line Increase Cellular ATP Levels. AAPS PharmSciTech.

[100] Kumar S., Karmacharya M., Michael I.J., Choi Y., Kim J., Kim I., Cho Y.-K. (2021). Programmed exosome fusion for energy generation in living cells. Nat. Catal..

[101] Pegoraro A., De Marchi E., Ferracin M., Orioli E., Zanoni M., Bassi C., Tesei A., Capece M., Dika E., Negrini M. (2021). P2X7 promotes metastatic spreading and triggers release of miRNA-containing exosomes and microvesicles from melanoma cells. Cell Death Dis..

[102] Enomoto T., Tanuma S., Yamada M. (1981). ATP Requirement for the Processes of DNA Replication in Isolated HeLa Cell Nuclei1. J. Biochem..

[103] D’Acunzo P., Pérez-González R., Kim Y., Hargash T., Miller C., Alldred M.J., Erdjument-Bromage H., Penikalapati S.C., Pawlik M., Saito M. (2021). Mitovesicles are a novel population of extracellular vesicles of mitochondrial origin altered in Down syndrome. Sci. Adv..

[104] Johannsen D.L., Ravussin E. (2009). The role of mitochondria in health and disease. Curr. Opin. Pharmacol..

[105] Moro L., Arbini A.A., Marra E., Greco M. (2008). Mitochondrial DNA depletion reduces PARP-1 levels and promotes progression of the neoplastic phenotype in prostate carcinoma. Cell. Oncol..

[106] Bianco F., Perrotta C., Novellino L., Francolini M., Riganti L., Menna E., Saglietti L., Schuchman E.H., Furlan R., Clementi E. (2009). Acid sphingomyelinase activity triggers microparticle release from glial cells. EMBO J..

